# Surviving Adulthood with Rare Combined Congenital Heart Defects: Complete AV Canal Defect, Ebstein’s Anomaly, and Right Ventricular Hypoplasia

**DOI:** 10.3390/life16020224

**Published:** 2026-01-29

**Authors:** Ana Peruničić, Stefan Veljković, Jovana Lakčević, Mirko Lipovac, Armin Šljivo, Slobodan Tomić, Milovan Bojić, Miloš Babić, Sanja Vučinić, Aleksandra Nikolić

**Affiliations:** 1 Cardiovascular Institute “Dedinje”, 11040 Belgrade, Serbia; 2 Department for Cardiosurgery, Clinical Center of University of Sarajevo, 71000 Sarajevo, Bosnia and Herzegovina; 3 Faculty of Medicine, University of Banja Luka, 78000 Banja Luka, Bosnia and Herzegovina; 4 Faculty of Medicine, University of Belgrade, 11000 Belgrade, Serbia

**Keywords:** Ebstein anomaly, atrioventricular septal defect, right ventricular hypoplasia, tricuspid regurgitation, congenital heart defects

## Abstract

**Background/Objectives.** Ebstein’s anomaly (EA), which accounts for fewer than 1% of congenital heart diseases, and atrioventricular canal defect (AVCD), present in approximately 4–5% of cases, exceptionally coexist, with this combination observed in fewer than 0.5% of patients with AVCD. We aim to report the oldest documented case of a 45-year-old female with the exceptionally rare combination of complete AVCD, EA, and right ventricular hypoplasia and to provide a concise review of these anomalies. **Case presentation.** Diagnosed in early childhood with a complete AVCD, pulmonary stenosis, and right ventricular (RV) hypoplasia, the patient underwent palliative surgical intervention with a modified Blalock–Taussig shunt at the age of 10 but did not receive subsequent regular follow-up. Over the ensuing 35 years, she remained largely untreated until presentation at 45 years of age with progressive exertional dyspnea, central cyanosis, and palpitations, corresponding to NYHA class III. Comprehensive multimodal imaging, including transthoracic echocardiography and cardiac magnetic resonance, revealed a complete AVCD with moderate-to-severe mitral regurgitation secondary to an anterior mitral leaflet cleft, severe tricuspid regurgitation, RV hypoplasia, and hallmark features of EA. Given the complex cardiac anatomy and the elevated surgical risk, the patient was considered inoperable, and a strategy of conservative management with multidisciplinary follow-up was implemented. **Conclusions.** This case highlights the exceptional longevity of a patient with the rare coexistence of complete AVCD, EA, and RV hypoplasia, surviving 45 years from diagnosis despite limited early intervention. It underscores the importance of lifelong follow-up in complex congenital heart disease and illustrates the role of multimodal imaging in assessing anatomy and guiding management when surgical options are high-risk or not feasible.

## 1. Introduction

### 1.1. Ebstein Anomaly: Clinical Spectrum, Hemodynamic Implications, and Associated Cardiac Defects

Ebstein anomaly (EA) is a rare congenital malformation, accounting for less than 1% of congenital heart disease cases, characterized by apical displacement of the septal and posterior tricuspid valve leaflets into the right ventricle (RV), resulting in atrialization of the RV inlet, severe tricuspid regurgitation, and a small functional RV [1]. The clinical spectrum of EA is broad: many patients present in infancy with cyanosis or heart failure, while others may remain asymptomatic into adulthood. Severity depends on the degree of leaflet displacement and the size of the functional RV [1].

EA is frequently associated with additional cardiac defects, with over 80% of patients exhibiting a secundum atrial septal defect (ASD) or patent foramen ovale that facilitates right-to-left shunting in the setting of tricuspid regurgitation [1]. Right ventricular outflow tract obstruction—for example pulmonary stenosis or atresia—is also common, especially in neonates. Less often, EA coexists with ventricular septal defects, tetralogy of Fallot, transposition of the great arteries, or even atrioventricular septal defects. These associated lesions compound the hemodynamic impact of EA. In addition, arrhythmias and accessory pathways (such as Wolff–Parkinson–White syndrome) are well known in EA patients, due to the markedly enlarged right atrium and displaced conduction tissue [1].

### 1.2. Atrioventricular Canal Defects: Pathophysiology, Genetic Associations, and Clinical Spectrum

Atrioventricular canal defect (AVCD), also known as an endocardial cushion or AV septal defect and accounting for approximately 4–5% of congenital cardiac malformations, arises from failed fusion of the superior and inferior endocardial cushions during development and, in its complete form, is characterized by a single common AV valve spanning both ventricles, an ostium primum ASD, and a large inlet ventricular septal defect. Partial and transitional forms of AVCD are less extensive, but all share atrial and AV valve anomalies [2,3]. AVCD are strongly linked to chromosomal abnormalities: roughly 40–50% of children with Down syndrome (trisomy 21) have an AVCD, and AVCD is rare outside of these syndromes. Other genetic syndromes (CHARGE, Ellis–van Creveld, Smith–Lemli–Opitz, heterotaxy, etc.) may also feature AVCD, reflecting the common developmental origin in endocardial cushion maldevelopment [2].

### 1.3. Coexistence of Ebstein Anomaly and Atrioventricular Canal Defect: Hemodynamic Complexity and Clinical Implications

Despite the descriptions above for each lesion individually, the combination of EA with AVCD is extremely uncommon. Large congenital heart databases show that EA is present in under 0.5% of patients with AVCD [2,3,4]. In practical terms, only a few dozen such cases have ever been reported. When both defects coexist, they create a highly complex physiology: there is right-to-left atrial shunting through the primum ASD (and any patent foramen ovale) in the setting of severe tricuspid regurgitation, plus volume overload of both ventricles from the common AV valve. The clinical picture in such patients is often dominated by neonatal cyanosis and heart failure, though severity can vary. Each defect influences the other—for example, low pulmonary blood flow from Ebstein’s regurgitation can accentuate hypoxemia, while the large left-to-right shunts of AVCD can impose further volume stress on the right atrium and ventricle [4,5].

### 1.4. Contemporary Medical and Interventional Management of Ebstein Anomaly Across the Lifespan

Ebstein anomaly management depends on age, symptom severity, and associated defects. In neonates and infants, initial therapy is mainly supportive: measures to reduce pulmonary vascular resistance (e.g., inhaled nitric oxide) and augment pulmonary blood flow (prostaglandin E_1_ to maintain the ductus) are used in cyanotic infants [1]. Milrinone is the inotrope of choice in cardiogenic shock because of its pulmonary vasodilatory effect, while catecholamines are avoided due to arrhythmia risk. Heart failure symptoms are managed with diuretics and afterload reduction, and atrial tachyarrhythmias are treated with rate-control or antiarrhythmic drugs (beta-blockers, procainamide, amiodarone, etc.). If tachyarrhythmias reoccur or are refractory, electrophysiology study and catheter ablation are often performed. In select cases, catheter-based interventions (for example, device closure of a secundum ASD or placement of a ductal stent) may be undertaken to improve circulation [1,6].

In ductal-dependent or severely cyanotic neonates, a palliative systemic-to-pulmonary shunt (e.g., modified Blalock–Taussig) or ductal stenting can augment pulmonary flow. Studies suggest that excessive delay of definitive repair until overt heart failure can worsen outcomes, so neonates meeting critical criteria should be considered early for surgical palliation or repair [1,6].

Older children and adults receive standard CHF therapy (diuretics, ACE inhibitors) for volume overload, as well as management of right-sided failure.

All patients are monitored for arrhythmias (Holter or event monitoring). Catheter ablation is strongly considered for recurrent accessory-pathway tachycardias or atrial flutter, especially before or during surgery [1,6].

### 1.5. Advanced Surgical Repair in Ebstein Anomaly and Associated Atrioventricular Canal Defects

Most patients with significant EA ultimately require surgery. Indications include right heart failure (NYHA class III–IV), progressive exercise intolerance, RV dysfunction or dilatation, paradoxical emboli, refractory arrhythmias, systemic desaturation (SaO_2_ < 90%), or severe tricuspid regurgitation [1,6,7]. In neonates, surgery is indicated for severe tricuspid regurgitation with cardiomegaly (>80% cardiothoracic ratio), PGE_1_-dependent pulmonary flow, or ventilation dependence. When EA is accompanied by an AVCD, the primum ASD and inlet VSD must also be repaired at the same time, and the common AV valve must be reconstructed carefully [1,6,7,8].

The current gold standard is the da Silva “cone” reconstruction, in which all tricuspid leaflets are mobilized, rotated, and sutured to form a 360° cone that coapts at the true annulus. This technique can be applied across a range of anatomies with good results [1,6,9]. Other repair methods include the Danielson plication (plicating the atrialized RV to reduce annular size and creating a competent monoleaflet valve) and the Carpentier monocusp repair (anterior leaflet mobilization with annular ring support). These repairs aim to eliminate regurgitation while preserving native tissue. When there is a large primum ASD (as in AV canal), patch closure of the ASD is performed concomitantly (unless it is being used as an intentional “pop-off” to decompress the right atrium) [1,6].

If the native valve is non-repairable—especially in older children or adults with severely dysplastic valves—tricuspid valve replacement may be necessary. Bioprosthetic valves are often preferred to avoid lifelong anticoagulation in young patients, but mechanical valves may be used in adults. Mechanical prostheses are avoided if RV dysfunction is severe, due to risk of thrombosis and poor leaflet motion [1,6]. Valve replacement eliminates regurgitation but carries long-term considerations of durability and anticoagulation [1,6].

In critically ill neonates, some surgeons use a one-and-a-half ventricle approach. This involves a bidirectional Glenn (superior cavopulmonary) shunt to unload the RV at the time of valve repair. In extreme cases with RV failure, a Starnes procedure (RV exclusion with systemic-to-pulmonary shunt) may be performed as a single-ventricle palliation, although this commits to a Fontan circulation thereafter [1,6].

Given the high incidence of atrial arrhythmias, a surgical Maze (or cryoablation) is often added during open surgery to ablate accessory pathways or atrial flutter circuits [1,6].

### 1.6. Guideline-Based Management Considerations for Ebstein Anomaly with Concomitant AV Canal Defect

No guidelines specifically address the combination of EA with AVCD, given its extreme rarity. However, management follows general principles for each lesion and expert consensus for complex cases. The American College of Cardiology/American Heart Association (ACC/AHA) 2018 congenital heart disease guidelines provide recommendations for EA [6]. They give a Class I recommendation for surgical repair in patients with significant tricuspid regurgitation accompanied by heart failure symptoms, worsening exercise capacity, or progressive RV dysfunction. Catheter ablation is strongly recommended (Class I) for patients with EA who have high-risk accessory pathways or multiple pathways. Electrophysiological study (with ablation if needed) is considered reasonable even in asymptomatic adults before tricuspid surgery because of the high prevalence of concealed pathways. A bidirectional Glenn shunt at the time of repair is a Class IIb (weak) consideration for patients with severe RV dilation/dysfunction when LV function is normal.

Regarding prophylaxis and anticoagulation, guidelines advise infective endocarditis prophylaxis for patients with unrepaired cyanotic disease, prosthetic valves, or prior endocarditis. Long-term anticoagulation is suggested for those with atrial fibrillation or prior paradoxical emboli [6].

Lastly, a 2024 consensus from congenital heart surgeons emphasizes that symptomatic neonates with EA require care in specialized centers. Some infants can be medically managed while pulmonary vascular resistance falls; however, those with critical cyanosis or heart failure should be moved quickly to surgical intervention (shunt or valve surgery). In practice, each child with combined EA and AVCD is managed on an individual basis by a multidisciplinary team, integrating techniques from both defect repairs [6].

## 2. Case Presentation

A 45-year-old woman was referred to the Center for Adult Congenital Heart Disease for evaluation of progressive dyspnea and recurrent palpitations. She had been diagnosed in early childhood with an AVCD accompanied by pulmonary artery stenosis and RV hypoplasia after presenting with central cyanosis. At 10 years of age, she underwent palliative surgical management with a modified right Blalock–Taussig shunt. However, she did not receive structured postoperative follow-up in the subsequent years.

According to the patient’s medical history, she delivered a healthy child by cesarean section in 2004 (at age 24) without any peripartum complications. In 2015, she was hospitalized at a regional center for suspected infective endocarditis; however, the diagnosis was excluded based on negative blood cultures and a transesophageal echocardiogram (TOE). She also reported having been treated for tuberculosis in 2020, although no medical documentation was available to substantiate this claim. Before the hospitalization in our institution, the patient remained largely stable, with mild exertional dyspnea corresponding to New York Heart Association (NYHA) class II. She had no hospitalizations for heart failure during this time.

At presentation, the patient demonstrated central cyanosis (SaO_2_ 87%), digital clubbing, and a systolic murmur most pronounced at Erb’s point and the interscapular region. Her symptoms corresponded to New York Heart Association (NYHA) functional class III.

Electrocardiography (ECG) demonstrated sinus rhythm at 71 bpm with right bundle branch block and signs of right ventricular hypertrophy, without evidence of acute ischemia.

Twenty-four-hour Holter showed sinus rhythm (average HR 73 bpm, range 50–148 bpm) with 343 ventricular ectopic beats (two morphologies, 10 couplets), 47 SVES, 2 couplets, and 4 short SVES runs (up to 11 beats), along with persistent IVCD and intermittent first-degree AV block at night. No sinus node dysfunction or pauses ≥2.0 s were observed. Seven-day telemetry confirmed dominant sinus rhythm (average HR 73 bpm, range 49–117 bpm) without significant bradycardia or pauses. Occasional SVES (≈90/24 h) and 37 short paroxysmal supraventricular tachycardia episodes (HR 106–167 bpm) were recorded. VESs were frequent (960–1250/24 h, two morphologies) with four couplets and occasional post-extrasystolic pauses. Intermittent first-degree AV block persisted; no ST changes or sustained atrial arrhythmias were noted. Mean BP: 115/73 mmHg.

Laboratory results revealed elevated red blood cell count (5.46 × 10^12^/L), hemoglobin (170 g/L), and NT-proBNP (698 pg/mL) levels.

During the 6 min walk test, oxygen saturation dropped from 89% to 78%. The Borg scale was 4, and the patient covered a distance of 560 m.

**Figure 1:**
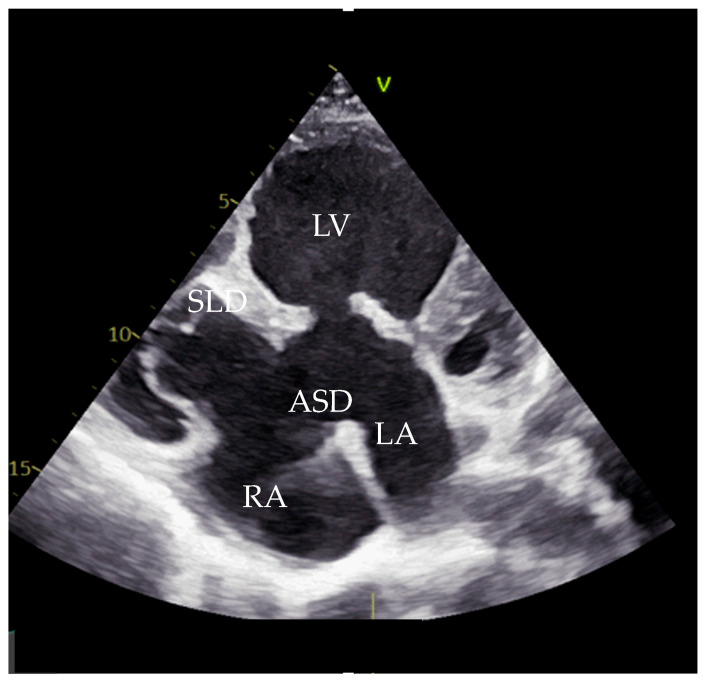
Echocardiography with apical four-chamber view showing apical displacement of tricuspid septal leaflet. LV—left ventricle, LA—left atrium, RA—right atrium, ASD—atrial septal defect, SLD—septal leaflet displacement.

**Figure 2:**
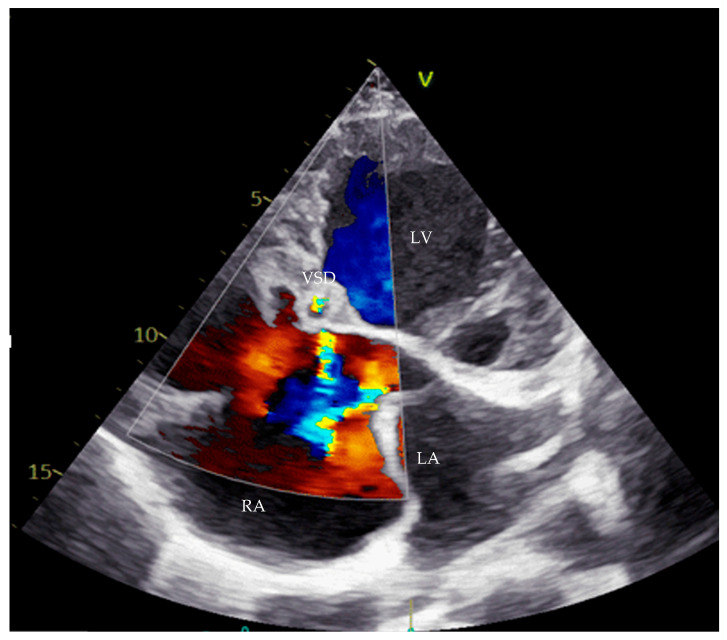
Echocardiography with apical four-chamber view showing VSD Gerbode type. LV—left ventricle, LA—left atrium, RA—right atrium, VSD—ventricular septal defect.

Transthoracic echocardiography (TTE) confirmed the diagnosis of a complete AVCD, with moderate to severe mitral regurgitation (MR) secondary to a cleft in the anterior mitral leaflet, a primum ASD, a ventricular septal defect, RV hypoplasia, and PS. Notably, apical displacement of the septal tricuspid leaflet by 19 mm and the presence of sail-like motion of the anterior tricuspid leaflet were highly suggestive of EA (Figure 1 and Figure 2).

Cardiac magnetic resonance imaging further confirmed the presence of a complete AVCD with a large primum defect measuring 35 mm, a small Gerbode-type VSD, and moderate to severe MR due to AML cleft. Severe tricuspid regurgitation was also observed, with a regurgitation fraction of 39%. The RV was hypoplastic, retaining only inlet and outlet portions. Features consistent with EA were described, including apical displacement of the septal tricuspid leaflet measured at 11 mm/m^2^. Pulmonary arteries were well-developed, with the right measuring 22 × 25 mm and the left 20 × 23 mm. The BT shunt was noted to be functional.

**Figure 3:**
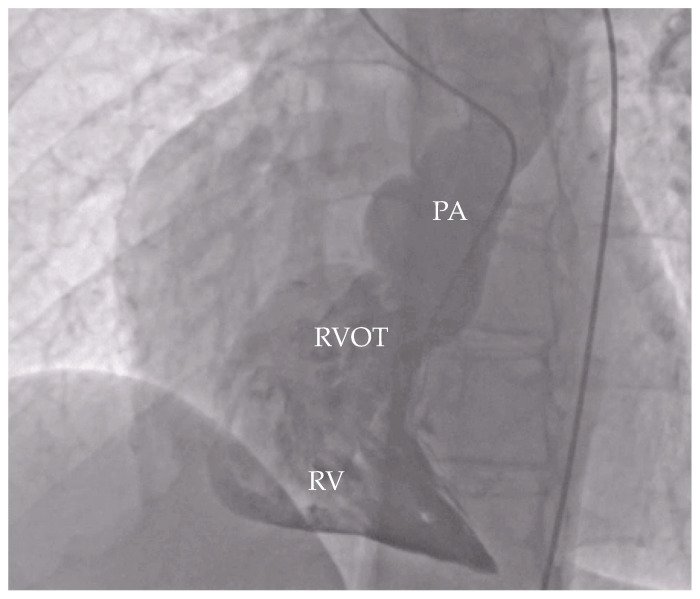
Angiography finding focusing on RV and RVOT. RV—right ventricle, RVOT—right ventricular outflow tract, PA—pulmonary artery.

Cardiac catheterization revealed normal pulmonary artery pressures (19/10/15 mmHg), a pulmonary vascular resistance (PVR) of 1.5 Wood units/m^2^, and a Qp/Qs ratio of 2.3 (Figure 3).

The patient was maintained on bisoprolol 5 mg once daily for rate control and furosemide 40 mg with potassium supplementation for management of volume overload, which decreased symptoms of NYHA III to NYHA II. ACE inhibitors, mineralocorticoid receptor antagonists, and SGLT2 inhibitors were not initiated due to specific clinical considerations and local availability at the time of treatment.

## 3. Discussion

To our knowledge, this case presents the oldest documented patient with a complete AVCD associated with EA—aged 45 years—significantly exceeding the previously reported oldest case of 20 years. To date, only 24 cases of this rare combination have been described in the literature, most of which were diagnosed postmortem, and the majority involved partial AVCD [10,11,12,13].

When EA and AVCD do coexist, the anatomy can be quite complex. The apical displacement of the tricuspid valve in EA may compound the AV valve defect of an AVCD and often leads to severe right-sided volume overload. Conversely, the large interatrial shunt of a complete AVCD can worsen cyanosis if the RV output is limited by EA-related RV hypoplasia or pulmonary obstruction. Hemodynamically, such patients may present as neonates with severe hypoxemia or heart failure, and the diagnosis may be challenging. The antenatal case series by Laforest et al. emphasizes that many historical diagnoses were made only at surgery or autopsy; in their report, detailed fetal echocardiography allowed prenatal identification of both EA and a partial AVCD [4].

Because the combination of EA and AVCD is so uncommon, there is no standardized surgical or management approach. Treatment typically requires addressing both lesions. In the published cases, outcomes have varied: some infants underwent tricuspid valve repair (cone procedure) with AV septal repair, whereas others died before intervention. Given the rarity of this combination, each case tends to be managed on an individual basis, often in specialized congenital heart centers.

Management of such patients is extremely challenging, even during the neonatal period, let alone in adulthood. In neonates, elevated pulmonary vascular resistance (PVR) poses a major risk, while in older patients, large septal defects such as primum ASD further compromise hemodynamics and complicate surgical decision-making [4].

In our case, therapeutic management was the subject of multiple in-depth discussions within the multidisciplinary heart team. In addition to having a complete form of AVCD, our patient also exhibited significant RV hypoplasia, which further limits surgical options. This combination of anomalies was first described by Guenthard in 1996 [14], who specifically highlighted RV hypoplasia as a major factor in deeming such patients inoperable.

The first attempted surgical repair of a complete AVCD associated with EA was reported by Fukuda et al. in 1999 [12], but the patient died three days postoperatively. Prior to that, six repairs had been performed in patients with partial or intermediate forms, with four survivors [13,15]. However, none of these cases involved the complete form seen in our patient. Other studies report similar findings [16,17]. In the few cases where the AVCD was partial or intermediate, surgical correction has sometimes succeeded. For example, Handler et al. reported full repair of a partial AVCD and EA in the 20-year-old patient [18]. More recently, Laforest et al. described antenatal diagnosis of partial AVCD + EA; after intensive neonatal care, the infant underwent a cone-type tricuspid repair and closure of the primum ASD at age 2 years, with good outcome [4]. In contrast, the combination of complete atrioventricular septal defect with EA has consistently been associated with poor outcomes, with no documented survivors following surgical repair reported in the literature.

Given the anatomical and hemodynamic complexity—particularly the combination of complete AVCD and RV hypoplasia—our patient, in line with previously reported outcomes, was considered high-risk and likely inoperable. In such cases, conservative management remains the most viable approach, focusing on optimal medical therapy, rhythm control, and close multidisciplinary follow-up.

This report describes a single patient, which inherently limits the generalizability of the findings. In addition, the therapeutic approach was predominantly conservative due to the patient’s high surgical risk; therefore, the outcomes observed may not be applicable to patients eligible for invasive or surgical management.

## 4. Conclusions

This case demonstrates that complex congenital cardiac anomalies, such as complete AVCD concomitant with EA, may remain hemodynamically stable over several decades. Multimodal imaging was pivotal in guiding clinical management: TTE enabled initial anatomical and functional assessment, while TOE, cardiac MRI, and CT angiography provided comprehensive evaluation of septal morphology, RV hypoplasia, valvular architecture, and associated lesions. These investigations informed the decision to pursue conservative therapy and facilitated individualized risk stratification. Long-term surveillance remains essential, as the patient may sustain relatively stable clinical status under optimized medical management.

## Data Availability

Data are available upon request.
